# Age-specific difference in the association between prediabetes and subclinical atherosclerosis: an analysis of a chinese prospective cohort study

**DOI:** 10.1186/s12933-022-01592-8

**Published:** 2022-08-10

**Authors:** Qiuyu Cao, Zhuojun Xin, Ruixin He, Tiange Wang, Min Xu, Jieli Lu, Meng Dai, Di Zhang, Yuhong Chen, Zhiyun Zhao, Shuangyuan Wang, Hong Lin, Weiqing Wang, Guang Ning, Yufang Bi, Yu Xu, Mian Li

**Affiliations:** 1 Department of Endocrine and Metabolic Diseases, Shanghai Institute of Endocrine and Metabolic Diseases, Ruijin Hospital, Shanghai Jiao Tong University School of Medicine, Shanghai, China; 2 Shanghai National Clinical Research Center for metabolic Diseases, Key Laboratory for Endocrine and Metabolic Diseases of the National Health Commission of the PR China, Shanghai Key Laboratory for Endocrine Tumor, State Key Laboratory of Medical Genomics, Ruijin Hospital, Shanghai Jiao Tong University School of Medicine, Shanghai, China

**Keywords:** Age, Prediabetes, Subclinical atherosclerosis, Temporal association

## Abstract

**Background:**

Prediabetes is an important risk factor of cardiovascular disease (CVD) and is associated with subclinical atherosclerosis. However, the evidence of prediabetes as a cardiovascular risk factor is mainly derived from middle-aged adults. Recently, multiple studies supported that prediabetes in older adults would not lead to higher risk of CVD or mortality. We aimed to investigate the age-specific difference in the association between prediabetes and subclinical atherosclerosis in a Chinese prospective cohort study.

**Methods:**

We included 4739 individuals aged ≥ 40 years and without diagnosed diabetes or CVD history, and divided them into middle-aged adults (age < 60) and older adults (age ≥ 60). Fasting plasma glucose (FPG), 2-h post-load plasma glucose (2 h-PPG) and glycated hemoglobin (HbA1c) were measured at baseline to identify prediabetes status. At follow-up visits, subclinical atherosclerosis status was assessed by branchial-ankle pulse wave velocity (baPWV) and carotid intima-media thickness (CIMT). Logistic regression analysis, restricted cubic splines and cross-lagged path analysis were used in statistical analysis.

**Results:**

1634 participants aged over 60 years, and 64.3% of them had prediabetes. 3105 participants aged 40–59 years, and 49.3% of them had prediabetes. We found that prediabetes was associated with increased risk of subclinical atherosclerosis in middle-aged adults, but the association attenuated substantially in older adults. Impaired glucose tolerance (IGT), compared to normal glucose tolerance, was associated with 39% lower risk of increased baPWV only in older adults. In accordance, the association between 2 h-PPG and risk of increased baPWV was “U-shaped” in older adults, while risk of elevated baPWV increased linearly with 2 h-PPG in middle-aged adults. In the cross-lagged analysis, increase in FPG and 2 h-PPG tended not to precede increase in baPWV in older adults, but appeared to increase simultaneously with baPWV in middle-aged ones.

**Conclusion:**

Our results indicated that prediabetes might be less related to subclinical atherosclerosis in older adults than in middle-aged adults and suggested that age was important to consider in the care of adults with prediabetes.

**Supplementary Information:**

The online version contains supplementary material available at 10.1186/s12933-022-01592-8.

## Background

The global burden of cardiovascular disease (CVD) has been increasing over decades [1]. Diabetes is a well-established risk factor for cardiovascular disease. Prediabetes, which is defined as impaired fasting glucose (IFG), impaired glucose tolerance (IGT) and raised glycated hemoglobin (HbA1c), has also been proved to be associated with CVD [2]. The prevalence of prediabetes has reached 50.1% in China in 2010 and this number increases substantially with age [3]. Despite the fact that more than half of the older adults have prediabetes, the cardiovascular risk of older adults attributed to prediabetes remains unclear and evidence is limited.

Most of our knowledge of prediabetes as a cardiovascular risk factor is derived from studies in middle-aged adults [4, 5]. Emerging evidence suggests that the strength of the association between glycemic status and cardiovascular outcomes may differ in older age compared with middle age [6]. A recent report from the Atherosclerosis Risk in Communities (ARIC) Study demonstrated that prediabetes diagnosed at 75 years was not associated with higher risk for cardiovascular mortality [7]. Evidence from the Lifetime Risk Pooling Project also suggested that prediabetes was associated with higher risk of heart failure in middle-aged Black women, but the association attenuated in older Black women [8]. These findings indicated that prediabetes may be of less importance to cardiovascular health in older adults than in middle-aged adults. However, the evidence was mainly derived from western population, and evidence from Chinese cohorts was lacking. Estimating the age-specific difference in the association between prediabetes and cardiovascular risk is essential to provide targeted glycemic screening strategy and appropriate treatment to middle-aged and older adults with prediabetes in China.

Since the onset of CVD events requires a long period of time, subclinical atherosclerosis diagnosed by elevated pulse wave velocity (PWV) or increased carotid intima-media thickness (CIMT) may allow better evaluation of the impact of prediabetes on the development of overt CVD within a relatively short period [9, 10]. PWV as a measure of arterial stiffness and CIMT as a measure of local vascular lesions are strong and early predictors of future CV events and all-cause mortality [11, 12]. The association between prediabetes and subclinical atherosclerosis in older adults is also unclear and requires more evidence from prospective cohorts.

Moreover, the associations between different prediabetes subtypes and CVD may be various. A Chinese nationwide cohort study supported that 2-hour post-load plasma glucose (2 h-PPG) better predicted CVD risk than fasting plasma glucose (FPG) or HbA1c [13]. Nevertheless, 2 h-PPG and HbA1c were seldomly included in the diagnosis criteria of prediabetes when estimating the association between prediabetes and CVD [6, 7].

Herein, using the established cutoffs for prediabetes recommended by American Diabetes Association (ADA) 2021 criteria, we compared the associations of prediabetes and glycemic metrics with subclinical atherosclerosis between middle-aged (40–59 years) and older adults (≥ 60 years), as well as the causal precedence between glycemic metrics and PWV by cross-lagged panel models in a Chinese prospective cohort.

## Methods

### Study population

It was a prospective cohort study of participants aged over 40 years from Jiading District in Shanghai, China. The recruitment and baseline evaluation were completed in 2010 and the follow-up examination was conducted in 2014. A detailed description of the study design, eligibility criteria and sampling has been published [14]. Among 10,375 individuals examined at baseline, we firstly excluded those who had diagnosed diabetes (n = 2000), had CVD (n = 206), did not complete baseline glycemic measurements or subclinical atherosclerosis detection (n = 381), leaving 7788 individuals for analysis. Then, we excluded those who did not complete follow-up glycemic measurements or subclinical atherosclerosis detection (n = 3049). Thus, a total of 4739 subjects were eventually included in the analysis with a median follow-up duration for 4.3 years. All of them did not take medication that could influence plasma glucose. In the additional analysis of CIMT, we included 4644 individuals who completed CIMT measurements at baseline and follow-up.

The study protocol was approved by the Institutional Review Board of Ruijin Hospital affiliated to the Shanghai Jiaotong University School of Medicine. Written informed consent was obtained from each participant.

### Baseline data collection

Face-to-face interviews were performed at baseline and in the follow-up period by trained personnel using a standard questionnaire to collect information on socioeconomic characteristics, lifestyle factors, medical history and current use of medications. Current smokers or drinkers were defined as those who had smoked cigarettes or consumed alcohol regularly in the past 6 months.

Measurements of body weight, height and waist circumstance were performed by trained nurses according to standard protocols. Body mass index (BMI) was calculated by dividing weight (kg) by height (m) squared. The blood pressure measurement was performed on the non-dominant arm, using an automated electronic device (OMRON Model HEM-752 FUZZY). Three blood pressure measurements were taken with participants in a seated position after 5 min of quiet rest and the mean value of three measurements was used in analysis.

All the participants were required to fast for ≥ 10 h before their visits and underwent a 75 g OGTT and blood samples were collected at 0 and 2 h during the test. FPG and 2 h- PPG were measured within 2 h after blood sample collection. HbA1c level was determined through High Performance Liquid Chromatography (HPLC) (BIO-RAD, Hercules, CA, USA). Total cholesterol, low density lipoprotein-cholesterol (LDL-C), high density lipoprotein-cholesterol (HDL-C), triglycerides (TG) and serum uric acid were measured using the chemiluminescence method with an auto-analyzer (Modular E170; Roche, Basel, Switzerland). Estimated glomerular filtration rate (eGFR) was assessed based on the Chronic Kidney Disease Epidemiology Collaboration formula for White/other (except Black) expressed in milliliters per minute per 1.73 m^2^: (1) females: Cr ≤ 0.7 mg/dL, eGFR = 144 × (Cr/0.7)^−0.329^ × (0.993) ^age^; Cr > 0.7 mg/dL, eGFR = 144 × (Cr/0.7)^−1.209^ × (0.993)^age^; and (2) males: Cr ≤ 0.9 mg/dL, eGFR = 141 × (Cr/0.9)^−0.411^ × (0.993)^age^; Cr > 0.9 mg/dL, eGFR = 141 × (Cr/0.9)^−1.209^ × (0.993)^age^. The Triglyceride–glucose index (TyG index) was calculated as ln [TG (mg/dL) × FPG (mg/dL)/2] [15].

All the participants underwent branchial-ankle pulse wave velocity (baPWV) measurements, with baPWV values determined by Colin VP-1000 (model BP203RPEII, form PWV/ABI; Omron Colin Medical Instruments, Tokyo, Japan). Briefly, participants attached cuffs around both arms and ankles after having rested for 10–15 min at room temperature (25 °C). Measurements from the brachial and tibial arteries were obtained simultaneously. Transit time, the time interval between the initial increase in brachial and tibial waveforms, and transit distance between the arm and ankle were measured. The value of baPWV was calculated as the transit distance divided by the transit time. We adopted the mean value of the right and left common baPWV for analysis. CIMT measurements were performed by an experienced sonographer using a high-resolution B-mode tomographic ultrasound system (Esaote Biomedica SpA, Italy) with a linear 7.5-MHz transducer. The CIMT was measured on the far wall of the right and left common carotid arteries, 1.5 cm proximal to the bifurcation. The distance between the leading edge of the first echogenic line and that of the second echogenic line at the end of diastole was calculated as the CIMT of either side. The larger value of the right and left CIMT was used for analysis.

### Definitions of prediabetes at baseline

In this population without diagnosed diabetes at baseline, we categorized participants according to ADA 2021 definitions for prediabetes based on FPG levels (100–125 mg/dL) and/or 2 h-PPG levels (140–199 mg/dL) and/or HbA1c levels (5.7–6.4%) [16]. We also defined prediabetes as having elevated HbA1c or impaired fasting glucose (IFG) or impaired glucose tolerance (IGT). Those whose FPG < 100 mg/dl and 2 h-PPG < 140 mg/dL and HbA1c < 5.7% were categorized to normal glucose regulation (NGR).

### Data collection at follow-up visit

In the follow-up examination, glucose parameters, baPWV and CIMT measurements were performed using the same protocol used during the baseline examination. The development of subclinical atherosclerosis was defined as new-onset increased baPWV or increased CIMT. We calculated the delta-baPWV as follow-up baPWV minus baseline baPWV, and new-onset increased baPWV was defined as having delta-baPWV over the 90th percentile. The increased CIMT was defined using the same method.

### Statistical analysis

Baseline characteristics were demonstrated according to the age and glycemic status. Continuous variables were presented as medians (interquartile ranges) and categorical variables were presented as numbers and proportions.

Odds ratios (ORs) and their corresponding 95% confidence intervals (CIs) for prediabetes status in relation to incident increased baPWV or incident increased CIMT in age groups were evaluated through multivariate logistic regression analyses. The ORs of prediabetes were calculated compared to normal glucose regulation. The ORs of IFG were calculated compared to normal FPG, and those of IGT were estimated compared to normal 2 h-PPG, and those of elevated HbA1c were calculated compared to normal HbA1c. Model 1 adjusted for age and sex, and Model 2 adjusted for age, sex, systolic blood pressure (SBP), diastolic blood pressure (DBP), BMI, TG, LDL-C, HDL-C, uric acid, eGFR, current smoking, drinking and anti-hypertensive drug. The interaction term between prediabetes status and age was used to obtain the P for interaction and the young-to-old ratio of odds ratios (RORs). To further evaluate the influence of sex, blood pressure, BMI, and TyG index on the age-specific associations, we performed logistic regression analyses in subgroups. The four subgroups were divided respectively by men or women, with hypertension or without hypertension, with BMI < 24 kg/m^2^ or with BMI ≥ 24 kg/m^2^, with TyG index < 4.64 (defined by the median of TyG index) or with TyG index ≥ 4.64.

Potential nonlinear associations between the levels of FPG, 2 h-PPG and HbA1c and the ORs of increased baPWV in different age groups were examined with restricted cubic splines [17]. Analyses adjusted for variables in Model 2, and 4 knots were located for each of the three glycemic measurements. Tests for nonlinearity, which compared a model containing only the linear term with a model containing the linear and restricted cubic spline terms were conducted by using likelihood ratio tests.

Repeated baPWV and glycemic measurements constituted a typical cross-lagged panel design [18, 19]. This design measured the effect size of baseline glycemic measurements on subsequent baPWV and the effect size of baseline baPWV on subsequent glycemic measurements simultaneously, adjusting for the auto-regressive effects. This analysis was performed in the total cohort and in age subgroups. Baseline and follow-up baPWV and glycemic measurements were adjusted for potential confounders before the cross-lagged analysis, including age, sex, SBP, DBP, BMI, TG, LDL-C, HDL-C, eGFR, uric acid, current smoking, drinking, and anti-hypertensive drug. We standardized both baPWV and glycemic measurements to means as 0 and SD as 1, because the ranges of the variates were different. Statistical difference in the effect size was examined using t test. Considering that those with abnormal glucose were excluded in the analysis, we performed a sensitivity analysis and further excluded those with baseline baPWV higher than the 95th percentile in the cross-lagged panel model.

## Results

### Age-specific baseline characteristics of prediabetes and NGR individuals

A total of 4739 participants without previous diabetes and cardiovascular diseases were included. 1634 participants aged over 60 years, and 1051 (64.3%) of them had prediabetes. 3105 participants aged 40–60 years, and 1532 (49.3%) of them had prediabetes (Table 1). Compared with the NGR individuals, those with prediabetes had higher level of baPWV, and higher BMI, waist circumstance, blood pressure, blood lipids and TyG index. The older adults had higher baPWV and worse metabolic profiles than the middle-aged adults.

**Table 1:** Baseline characteristics of participants according to age and glycemic status

	People aged ≥ 60 years		People aged < 60 years	
	Prediabetes (n = 1051)	NGR (n = 583)	Prediabetes (n = 1532)	NGR (n = 1573)
Age, years	65.5 (62.5–69.4)	64.9 (62.1–69.5)	53.9 (49.8–56.9)	51.9 (47.2–55.9)
Male, n (%)	395 (37.6)	255 (43.7)	486 (31.7)	524 (33.3)
baPWV, cm/s	1688 (1512–1946)	1648 (1449–1901)	1463 (1310–1627)	1375 (1257–1542)
FPG, mmol/L	5.29 (4.91–5.68)	4.91 (4.61–5.16)	5.27 (4.86–5.72)	4.84 (4.55–5.12)
2 h-PPG, mmol/L	7.83 (6.37–8.92)	6.07 (5.19–6.87)	7.12 (5.83–8.48)	5.73 (4.92–6.52)
HbA1c, %	5.80 (5.60-6.00)	5.40 (5.30–5.50)	5.80 (5.60–5.90)	5.40 (5.20–5.50)
SBP, mmHg	147.7 (134.3-160.3)	143.3 (130.3-157.7)	136.7 (125.3-149.7)	130.7 (119.7-143.3)
DBP, mmHg	82.0 (76.0–89.0)	81.3 (75.0-87.7)	84.3 (77.0–91.0)	81.7 (74.7–89.0)
BMI, kg/m^2^	25.1 (23.1–27.3)	24.4 (22.5–26.8)	25.0 (22.9–27.2)	24.1 (22.1–26.1)
Waist circumference, cm	83.0 (77.5–89.0)	82.0 (75.5–88.0)	82.0 (76.0–88.0)	80.0 (74.0-85.5)
Triglyceride, mmol/L	1.45 (1.00-1.95)	1.24 (0.90–1.66)	1.41 (0.99–2.10)	1.24 (0.90–1.73)
LDL-C, mmol/L	3.28 (2.77–3.84)	3.11 (2.60–3.63)	3.17 (2.72–3.78)	3.00 (2.50–3.50)
HDL-C, mmol/L	1.32 (1.12–1.56)	1.35 (1.12–1.60)	1.28 (1.10–1.54)	1.33 (1.11–1.59)
Uric acid, umol/L	297.4 (244.2-360.8)	289.5 (235.9-359.5)	282.1 (228.7-344.8)	266.7 (220.8-332.3)
eGFR, mL/(min 1.73m^2^)	93.3 (86.4–97.5)	92.6 (86.4–97.0)	103.5 (98.8-108.2)	104.6 (99.5-109.3)
TyG index	4.69 (4.51–4.86)	4.58 (4.42–4.73)	4.68 (4.51–4.89)	4.58 (4.41–4.75)
Current smoking, n (%)	179 (17.6)	102 (18.1)	295 (19.9)	339 (22.4)
Current drinking, n (%)	106 (10.3)	64 (11.2)	153 (10.3)	147 (9.8)
Antihypertensive drugs, n (%)	399 (38.0)	142 (24.4)	363 (23.7)	263 (16.7)

NGR, normal glucose regulation; BaPWV, branchial-ankle pulse wave velocity; FPG, fasting plasma glucose; 2 h-PPG, 2 h-postload plasma glucose; HbA1c, glycated hemoglobin; SBP, systolic blood pressure; DBP, diastolic blood pressure; BMI, body mass index; LDL-C, low-density lipoprotein cholesterol; HDL-C, high-density lipoprotein cholesterol; eGFR, estimated glomerular filtration rate; TyG index, triglyceride–glucose index

### The association between prediabetes and increased baPWV and CIMT in middle-aged and older adults

As suggested in Table 2, after adjusted for age and sex, baseline prediabetes was associated with 44% higher risk of developing increased baPWV in those aged < 60 years (OR = 1.44, 95% CI 1.07–1.94), whereas prediabetes was not significantly associated with increased baPWV in those aged over 60 years (OR = 0.82, 95% CI 0.63–1.08). After fully-adjusted for age, sex, SBP, DBP, BMI, TG, LDL-C, HDL-C, uric acid, eGFR, smoking and drinking habits and anti-hypertensive drug, baseline prediabetes was associated with 36% higher risk of increased baPWV in the middle-aged adults (OR = 1.36, 95% CI 1.00–1.86), and the association between prediabetes and increased baPWV was not significant in the older individuals (OR = 0.89, 95% CI 0.67–1.18). The risk of increased baPWV attributed to prediabetes was higher in the middle-aged adults than in the older ones (Young-to-old ROR = 1.87, 95% CI 1.25–2.81) with P for interaction = 0.003. The risk of increased baPWV contributed by IFG was also higher in the middle-aged subjects (OR = 1.71, 95% CI 1.20–2.44) than in the older adults (OR = 0.98, 95% CI 0.70–1.39). Notably, in the older adults, baseline IGT was associated with significantly lower risk of developing increased baPWV (OR = 0.61, 95% CI 0.44–0.83), while IGT in those aged < 60 years was associated with higher risk of increased baPWV (OR = 1.31, 95% CI 0.92–1.86), with P for interaction < 0.001. The risk of increased baPWV attributable to elevated HbA1c was higher in the middle-aged adults than in the older ones, with P for interaction = 0.067. Besides, as shown in Additional file 1: Table S1, the risk of increased CIMT attributable to baseline IFG or IGT was significantly higher in the middle-aged subjects than in the older ones, with P for interaction = 0.023 and P for interaction = 0.006.

**Table 2:** The association between overall prediabetes, IFG, IGT and elevated HbA1c and increased baPWV in middle-aged and old adults

	Age < 60 years		Age ≥ 60 years		P for interaction	Young:Old ROR
	OR (95% CI) in Model 1	OR (95% CI) in Model 2	OR (95% CI) in Model 1	OR (95% CI) in Model 2		
NGR	1.00	1.00	1.00	1.00	–	–
Prediabetes	1.44 (1.07–1.94)	1.36 (1.00-1.86)	0.82 (0.63–1.08)	0.89 (0.67–1.18)	0.003	1.87 (1.25–2.81)
IFG	1.64 (1.16–2.31)	1.71 (1.20–2.44)	0.95 (0.68–1.33)	0.98 (0.70–1.39)	0.006	1.99 (1.22–3.23)
IGT	1.30 (0.94–1.81)	1.31 (0.92–1.86)	0.59 (0.44–0.80)	0.61 (0.44–0.83)	< 0.001	2.67 (1.70–4.20)
Elevated HbA1c	1.46 (1.09–1.95)	1.38 (1.02–1.87)	1.04 (0.80–1.36)	1.13 (0.86–1.48)	0.067	1.45 (0.98–2.16)

The ORs of prediabetes were calculated compared to normal glucose regulation. The ORs of IFG were calculated compared to normal FPG, and those of IGT were estimated compared to normal 2 h-PPG, and those of elevated HbA1c were calculated compared to normal HbA1c. Model 1: adjust for age and sex; Model 2: adjust for age, sex, SBP, DBP, BMI, TG, LDL-C, HDL-C, uric acid, eGFR, smoking, drinking and anti-hypertensive drug

OR, odds ratio; CI, confidence interval; ROR, ratio of odds ratio; baPWV, branchial-ankle pulse wave velocity; NGR, normal glucose regulation; IFG, impaired fasting glucose; IGT, impaired glucose tolerance; HbA1c, glycated hemoglobin; SBP, systolic blood pressure; DBP, diastolic blood pressure; BMI, body mass index; TG, triglyceride; LDL-C, low-density lipoprotein cholesterol; HDL-C, high-density lipoprotein cholesterol; eGFR, estimated glomerular filtration rate

We also analyzed the age-specific association between prediabetes and increased baPWV in adults with different sex, hypertensive status, or obesity status, or TyG index. After full adjustment, the age-specific difference in the association between prediabetes and increased baPWV was more significant in men than in women, with P for interaction = 0.007 and P for interaction = 0.115 (Additional file 1: Table S2). The age-specific difference in the association of prediabetes with increased baPWV was similar between the normotensive and hypertensive adults (Additional file 1: Table S3). In the overweight and obese adults, the age-specific difference in the association of prediabetes with risk of increased baPWV was more obvious than in the normal-weight adults, with P for interaction = 0.054 and P for interaction = 0.146 (Additional file 1: Table S4). In those whose TyG index ≥ 4.64, the age-specific difference was more significant than in those whose TyG index < 4.64, with P for interaction = 0.002 and P for interaction = 0.374 (Additional file 1: Table S5).

### The association between FPG, 2 h-PPG and HbA1c and risk of increased baPWV in middle-aged and older adults

**Fig. 1:**
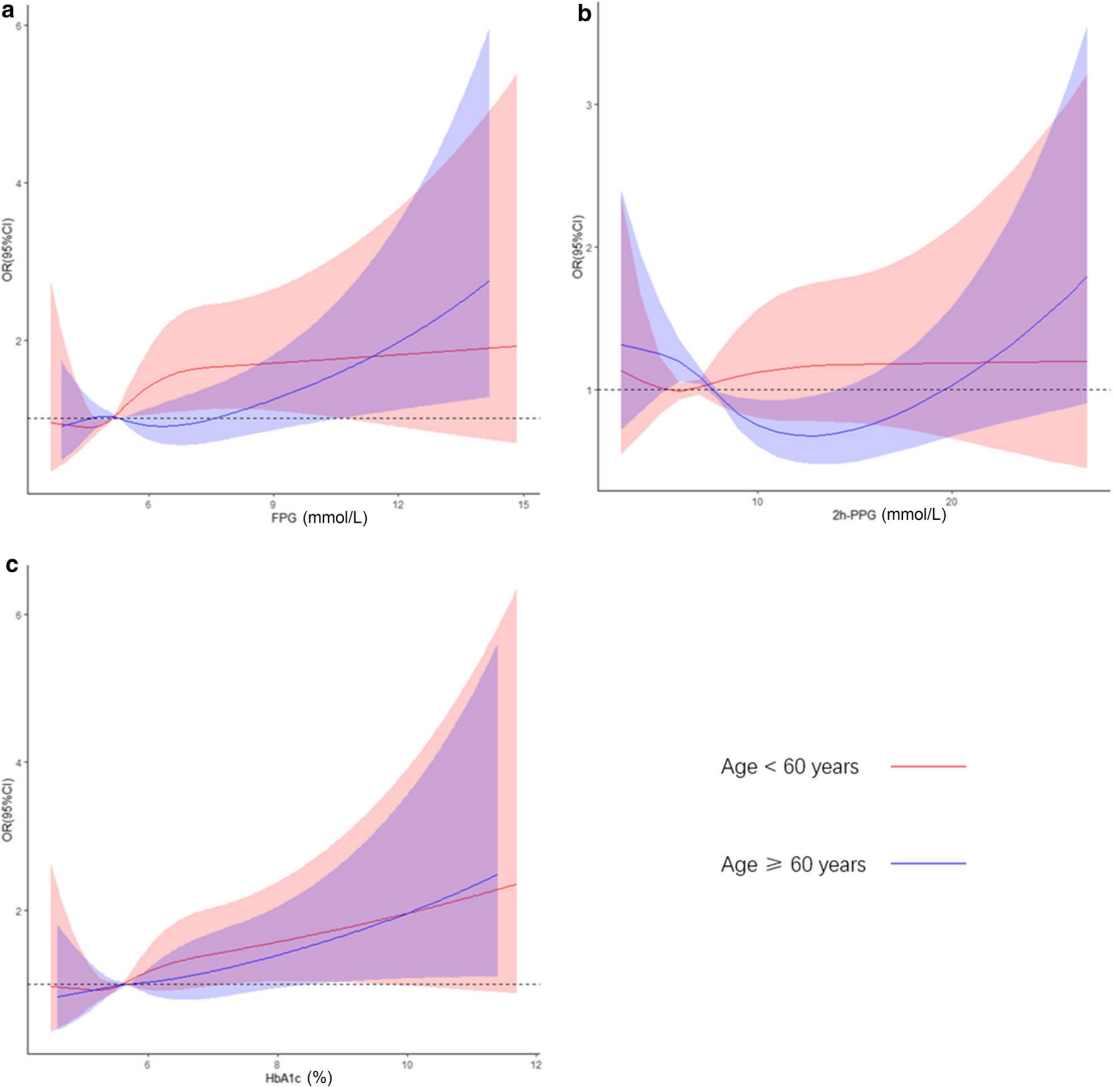
The age-specific associations between glycemic measurements and OR (95% CI) for elevated baPWV. ** a** FPG; **b** 2 h-PPG; **c** HbA1c. OR, odds ratio; CI, confidence interval; baPWV, branchial-ankle pulse wave velocity; FPG, fasting plasma glucose; 2 h-PPG, 2 h-postload plasma glucose; HbA1c, glycated hemoglobin

As shown in Fig. 1, multivariable-adjusted restricted cubic spline analyses suggested “U-shaped” associations of 2 h-PPG with risk of increased baPWV in older adults aged over 60 years (P for nonlinearity = 0.004), but indicated a linear relationship between 2 h-PPG and risk of increased baPWV in middle-aged individuals (P for nonlinearity = 0.904). When 2 h-PPG was in the range of IGT (between 7.8 and 11.1 mmol/L), the risk of increased baPWV was higher in the middle-aged adults than in the older adults. The risk of elevated baPWV increased linearly with FPG and HbA1c in older adults, and the associations were not significantly different between older adults and middle-aged subjects.

### Cross-lagged analyses for FPG, 2 h-PPG, HbA1c and baPWV in middle-aged and older adults

In the older adults aged over 60 years, after fully adjusted for age, sex, SBP, DBP, BMI, TG, LDL-C, HDL-C, eGFR, uric acid, smoking and drinking status, and use of anti-hypertensive drug, the standardized correlation coefficient of baseline FPG and follow-up baPWV (β1) was 0.00 (95% CI − 0.02 to 0.02), but that of baseline baPWV and follow-up FPG (β2) was 0.06 (95% CI 0.04–0.08), which was significantly higher than β1 (P = 0.017), while in the middle-aged adults, the standardized correlation coefficient of baseline FPG and follow-up baPWV (β3) was 0.03 (95% CI 0.02–0.04), and that of baseline baPWV and follow-up FPG (β4) was 0.00 (95% CI − 0.02 to 0.02), which was lower than β3 (P = 0.090). These results indicated that increase in FPG tended not to precede increase in baPWV in older adults, but FPG and baPWV appeared to increase simultaneously in middle-aged ones (Table 3).

**Table 3:** Adjusted cross-lagged standard regression coefficient of baPWV and FPG

	R^2^ of PWV	R^2^ of FBG	baPWV1 → baPWV2	FPG1 → FPG2	FPG1 → baPWV2	baPWV1 → FPG2	P value*
Age ≥ 60	0.58	0.30	0.72 (0.70–0.74)	0.52 (0.50–0.54)	0.00 (− 0.02 to 0.02)	0.06 (0.04–0.08)	0.017
Age < 60	0.53	0.34	0.59 (0.58–0.60)	0.55 (0.54–0.56)	0.03 (0.02–0.04)	0.00 (− 0.02 to 0.02)	0.090

Model is adjusted for baseline age (quartile), sex, systolic blood pressure (quartile), diastolic blood pressure (quartile), body mass index (quartile), triglyceride (quartile), low-density lipoprotein cholesterol (quartile), high-density lipoprotein cholesterol (quartile), estimated glomerular filtration rate (quartile), uric acid (quartile), current smoking, current drinking, and anti-hypertensive drug. The P value is calculated by t-test of the two cross-lagged standard regression coefficients of baPWV and FPG

BaPWV, branchial-ankle pulse wave velocity; FPG, fasting plasma glucose

Cross-lagged analysis for 2 h-PPG and baPWV showed that, increase in baPWV preceded increase in 2 h-PPG (P = 0.001) in older adults, but baPWV and 2 h-PPG increased simultaneously (P = 0.090) in middle-aged adults (Table 4). And cross-lagged analysis for HbA1c and baPWV indicated that baPWV and HbA1c increased simultaneously in both older and middle-aged adults (Table 5). In the sensitivity analysis, the results remained similar (Additional file 1: Tables S6–S8).

**Table 4:** Adjusted cross-lagged standard regression coefficient of baPWV and 2 h-PPG

	R^2^ of PWV	R^2^ of PPG	baPWV1→baPWV2	PPG1→PPG2	PPG1→baPWV2	baPWV1→PPG2	P value*
Age ≥ 60	0.58	0.29	0.72 (0.70–0.74)	0.46 (0.44–0.48)	− 0.03 (− 0.05 to (− 0.01))	0.06 (0.04–0.08)	0.001
Age < 60	0.53	0.27	0.59 (0.58–0.60)	0.46 (0.44–0.48)	0.00 (− 0.01 to 0.01)	0.03 (0.01–0.05)	0.090

Model is adjusted for baseline age (quartile), sex, systolic blood pressure (quartile), diastolic blood pressure (quartile), body mass index (quartile), triglyceride (quartile), low-density lipoprotein cholesterol (quartile), high-density lipoprotein cholesterol (quartile), estimated glomerular filtration rate (quartile), uric acid (quartile), current smoking, current drinking, and anti-hypertensive drug. The P value is calculated by t-test of the two cross-lagged standard regression coefficients of baPWV and 2 h-PPG

BaPWV, branchial-ankle pulse wave velocity; 2 h-PPG, 2 h-postload plasma glucose

**Table 5:** Adjusted cross-lagged standard regression coefficient of baPWV and HbA1c

	R^2^ of PWV	R^2^ of HbA1c	baPWV1 → baPWV2	HbA1c1 → HbA1c2	HbA1c1 → baPWV2	baPWV1 → HbA1c2	P value*
Age ≥ 60	0.58	0.37	0.72 (0.70–0.74)	0.57 (0.55–0.59)	0.04 (0.02–0.06)	0.07 (0.05–0.09)	0.145
Age < 60	0.53	0.34	0.59 (0.58–0.60)	0.54 (0.53–0.55)	0.03 (0.02–0.04)	0.00 (-0.02-0.02)	0.090

Model is adjusted for baseline age (quartile), sex, systolic blood pressure (quartile), diastolic blood pressure (quartile), body mass index (quartile), triglyceride (quartile), low-density lipoprotein cholesterol (quartile), high-density lipoprotein cholesterol (quartile), estimated glomerular filtration rate (quartile), uric acid (quartile), current smoking, current drinking, and anti-hypertensive drug. The P value is calculated by t-test of the two cross-lagged standard regression coefficients of baPWV and HbA1c

BaPWV, branchial-ankle pulse wave velocity; HbA1c, glycated hemoglobin

## Discussion

In the prospective analysis of 4739 Chinese adults aged 40 years and over, prediabetes was associated with increased risk of subclinical atherosclerosis in middle-aged adults, but the association attenuated substantially in older adults. IGT, compared to normal glucose tolerance, was associated with 39% lower risk of increased baPWV in older adults. In accordance, the association between 2 h-PPG and risk of increased baPWV was “U-shaped” in older adults, while risk of elevated baPWV increased linearly with 2 h-PPG in middle-aged adults. In the cross-lagged path analysis, increase in glycemic parameters tended not to precede increase in baPWV in older adults, but glycemic parameters and baPWV appeared to increase simultaneously in middle-aged ones. Taken together, our results indicated that prediabetes may be less related to subclinical atherosclerosis in older adults than in middle-aged adults.

Currently, evidence for the cardiovascular risk associated with prediabetes in older adults is limited, but it was reported recently that prediabetes diagnosed by FPG 100–125 mg/dL was not associated with CVD events or mortality in older adults [6]. Evidence from the ARIC study and the Lifetime Risk Pooling Project both supported that the association between prediabetes and cardiovascular mortality or heart failure was not significant in older individuals [7, 8]. Recently, in a male-prominent Chinese cohort aged over 75 years, it was also demonstrated that prediabetes was not associated with future risk for CVD (HR = 1.17, 95% CI 0.82–1.69) [20]. However, since the onset of CVD requires a long period of time, the association between prediabetes and CVD events in older adults may attenuate due to the relatively short period of follow-up. Therefore, using subclinical atherosclerosis, the earlier sign of CVD events as the surrogate outcome, could detect the effect of prediabetes on cardiovascular risk more sensitively.

It was reported previously that an increase in FPG within the normal range was associated with aggravated arterial stiffness in a cross-sectional study [21]. Another cross-sectional analysis of the ARIC study reported that prediabetes was associated with higher baPWV among older adults [22]. But evidence of age-specific associations from prospective studies is lacking. Our results demonstrated that, prediabetes identified by FPG 100–125 mg/dL or 2 h-PPG 160–199 mg/dL or HbA1c 5.7−6.4% was associated with excess risk of subclinical atherosclerosis estimated by increased baPWV in middle-aged adults aged 40–59 years, but the association disappeared in older adults aged over 60 years. Moreover, the risk of increased CIMT associated with two subtypes of prediabetes, IFG and IGT, was significantly higher in middle-aged subjects than in older ones. These results further supported that prediabetes was of less importance to early cardiovascular risk in older adults than in middle-aged adults. It was reported that onset of prediabetes earlier in life might be an important cardiovascular risk factor in this group that might reflect underlying insulin resistance, while onset of prediabetes later in life might be a more benign manifestation of aging that did not increase the risk [8]. Accordingly, in our subgroup analysis, we found that hypertension status would not influence the age-specific difference, but obesity status and insulin resistance level estimated by TyG index would influence the results, indicating that different body composition or insulin resistance in middle-aged and older adults might contribute to the different relations between prediabetes and subclinical atherosclerosis. In addition, we found that the age-specific difference was more obvious in men than in women. It was possible that a larger proportion of males were obese or had high TyG index than females. The mechanisms behind age-specific differences still require further investigation.

The recommendations for screening, diagnosis and management of prediabetes in older adults are largely based on conclusions from the general adult population, which mainly includes middle-aged adults. Current ADA guidelines recommended that adults with prediabetes be screened every year for diabetes and be referred to a lifestyle intervention to encourage weight loss [16]. Nevertheless, the diabetes prevention study PREVIEW, which included younger, middle-aged and older adults, found that older adults benefited less from a lifestyle intervention in relation to cardiometabolic health markers than younger adults, despite greater weight loss [23]. The findings of the current study support less aggressive treatment to cardiovascular disease prevention in older adults with prediabetes than in middle-aged ones with prediabetes, given the low risk of subclinical atherosclerosis among older adults with prediabetes in this study.

A previous report from a Chinese nationwide cohort study demonstrated that different glycemic measurements, including FPG, 2 h-PPG and HbA1c, might have various predictive value of CVD events, and 2 h-PPG had stronger association with CVD compared to FPG and HbA1c in Chinese [13]. This study also reported that the association between 2 h-PPG and cardiovascular disease attenuated in individuals aged ≥ 60 years (P for interaction < 0.001). We found that IGT was associated with 39% lower risk of elevated baPWV in older adults, while IGT was not significantly associated with elevated baPWV in middle-aged adults (P for interaction < 0.001). Besides, our restricted cubic spline analysis also observed a “U-shaped” association between 2 h-PPG and risk of increased baPWV only in older adults. These results indicated that the association between 2 h-PPG and subclinical atherosclerosis could be influenced by age. Further investigations were required to evaluate the risk of cardiovascular events in older adults with IGT.

It is widely believed that diabetes contributed to subclinical atherosclerosis [24]. However, the association between subclinical atherosclerosis and diabetes might be bi-directional according to results of previous studies [25, 26]. A recent report from the Kailuan study in China demonstrated that increase of baPWV appeared to precede the increase in FPG in cross-lagged path analysis [19]. We performed path analysis in middle-aged and older adults respectively and found that the path from baseline baPWV to subsequent FPG was significantly stronger than the other path from baseline FPG to subsequent baPWV in older adults, and the paths were varied in middle-aged adults. The cross-lagged relationship between 2 h-PPG, HbA1c and baPWV was similar to that of FPG in the two age groups. These results supported age-specific difference in the direction of association between glycemic parameters and subclinical atherosclerosis. And for the older adults, increased glycemic measurements might not precede subclinical atherosclerosis. Further analysis on the causal relationship between diabetes and subclinical atherosclerosis in people of different age is required.

The strength of this study includes the large, community-based study population with comprehensive assessment of glycemic status, including FPG, 2 h-PPG and HbA1c, and careful identification of subclinical atherosclerosis by baPWV and CIMT.

This study also has limitations. Firstly, the study only recruited adults aged over 40 years, and the association between prediabetes and subclinical atherosclerosis in younger adults requires more investigation. Secondly, the cohort was followed up for a relatively short period (4.3 years), and the age-specific difference in the associations could be more significant in the long term. Thirdly, we carefully controlled for confounders in the analyses, but biases from unmeasured confounding and reverse causality might exist. Finally, selective survival bias before study enrolment could attenuate estimated effects of prediabetes on cardiovascular risk in older adults. Therefore, the generalizability of our findings should be interpreted with caution.

## Conclusion

In summary, prediabetes identified by FPG, 2 h-PPG and HbA1c was not associated with subclinical atherosclerosis in older adults, but was associated with subclinical atherosclerosis in middle-aged adults. Increase in FPG and 2 h-PPG tended not to precede increase in baPWV in older adults, but appeared to increase simultaneously with baPWV in middle-aged ones. Our results confirm that age is important to consider in the care of adults with prediabetes, since prediabetes may be less related to subclinical atherosclerosis in older adults than in middle-aged adults.

## Supplementary Information

**Additional file 1.** This additional file provided Additional tables in the paper. 

## Data Availability

The data are available from the corresponding authors upon reasonable request.

## Abbreviations

2 h-PPG: 2-h post-load plasma glucose
ADA: American Diabetes Association
ARIC: Atherosclerosis Risk in Communities
BaPWV: Branchial-ankle pulse wave velocity
BMI: Body mass index
CI: Confidence interval
CIMT: Carotid intima-media thickness.
CVD: Cardiovascular diseases
DBP: Diastolic blood pressure
eGFR: Estimated glomerular filtration rate
FPG: Fasting plasma glucose
HbA1c: Glycated hemoglobin
HDL-C: High-density lipoprotein cholesterol
HPLC: High performance liquid chromatography
IFG: Impaired fasting glucose
IGT: Impaired glucose tolerance
LDL-C: Low-density lipoprotein cholesterol
NGR: Normal glucose regulation
OR: Odds ratio
ROR: Ratio of odds ratio
SBP: Systolic blood pressure
TG: Triglycerides
TyG index: Triglyceride–glucose index
WC: Waist circumstance
