# Supplementary material for: Age-specific difference in the association between prediabetes and subclinical atherosclerosis: an analysis of a chinese prospective cohort study
Source: Cardiovasc Diabetol. 2022 Aug 10;21:153. doi: 10.1186/s12933-022-01592-8 (PMC9364510; doi:10.1186/s12933-022-01592-8)
Supplement: Supplementary file 1 — Additional file 1. This additional file provided Additional tables in the paper. [file 12933_2022_1592_MOESM1_ESM.docx]

**Additional file 1**

Table S1. The association between overall prediabetes, IFG, IGT and elevated HbA1c and increased CIMT in middle-aged and old adults

|  | Age <60 years | | Age ≥60 years | | P for interaction | Young: Old ROR |
| --- | --- | --- | --- | --- | --- | --- |
|  | OR (95%CI) in Model 1 | OR (95%CI) in Model 2 | OR (95%CI) in Model 1 | OR (95%CI) in Model 2 |  |  |
| NGR | 1.00 | 1.00 | 1.00 | 1.00 | - | - |
| Prediabetes | 1.10 (0.91-1.33) | 1.11 (0.91-1.36) | 0.77 (0.60-1.00) | 0.86 (0.66-1.13) | 0.054 | 1.37 (0.99-1.90) |
| IFG | 1.12 (0.87-1.44) | 1.09 (0.84-1.41) | 0.64 (0.45-0.90) | 0.68 (0.48-0.97) | 0.023 | 1.65 (1.07-2.55) |
| IGT | 1.34 (1.07-1.68) | 1.37 (1.08-1.73) | 0.80 (0.61-1.06) | 0.89 (0.67-1.18) | 0.006 | 1.64 (1.15-2.35) |
| Elevated HbA1c | 1.00 (0.82-1.22) | 1.00 (0.81-1.22) | 0.95 (0.74-1.23) | 0.99 (0.76-1.28) | 0.778 | 1.05 (0.76-1.45) |

In the analysis of CIMT, we included 4644 individuals who completed CIMT measurements at baseline and follow-up. The ORs of prediabetes were calculated compared to normal glucose regulation. The ORs of IFG were calculated compared to normal FPG, and those of IGT were estimated compared to normal 2h-PPG, and those of elevated HbA1c were calculated compared to normal HbA1c. Model 1: adjust for age and sex; Model 2: adjust for age, sex, SBP, DBP, BMI, TG, LDL-C, HDL-C, uric acid, eGFR, smoking, drinking and anti-hypertensive drug. OR, odds ratio; CI, confidence interval; ROR, ratio of odds ratio; CIMT, carotid intimal media thickness; NGR, normal glucose regulation; IFG, impaired fasting glucose; IGT, impaired glucose tolerance; HbA1c, glycated hemoglobin; SBP, systolic blood pressure; DBP, diastolic blood pressure; BMI, body mass index; TG, triglyceride; LDL-C, low-density lipoprotein cholesterol; HDL-C, high-density lipoprotein cholesterol; eGFR, estimated glomerular filtration rate.

Table S2. The association between overall prediabetes, IFG, IGT and elevated HbA1c and increased baPWV in adults with different age and sex

|  | Men | | | Women | | |
| --- | --- | --- | --- | --- | --- | --- |
|  | Age <60 years (n=1010) | Age ≥60 years (n=650) | P for interaction | Age <60 years (n=2095) | Age ≥60 years (n=984) | P for interaction |
| NGR | 1.00 | 1.00 | - | 1.00 | 1.00 | - |
| Prediabetes | 1.69 (0.97-2.95) | 0.73 (0.46-1.15) | 0.007 | 1.18 (0.81-1.72) | 0.98 (0.68-1.42) | 0.115 |
| IFG | 2.26 (1.26-4.06) | 0.93 (0.53-1.63) | 0.025 | 1.45 (0.92-2.29) | 1.00 (0.64-1.57) | 0.084 |
| IGT | 2.63 (1.44-4.81) | 0.43 (0.24-0.78) | <0.001 | 0.95 (0.61-1.48) | 0.69 (0.47-1.02) | 0.047 |
| Elevated HbA1c | 1.31 (0.77-2.26) | 0.95 (0.60-1.49) | 0.175 | 1.34 (0.93-1.94) | 1.23 (0.87-1.75) | 0.278 |

Models were adjusted for age, BMI, TG, LDL-C, HDL-C, uric acid, eGFR, smoking, drinking and anti-hypertensive drug. OR, odds ratio; CI, confidence interval; ROR, ratio of odds ratio; baPWV, branchial-ankle pulse wave velocity; IFG, impaired fasting glucose; IGT, impaired glucose tolerance; HbA1c, glycated hemoglobin; SBP, systolic blood pressure; DBP, diastolic blood pressure; BMI, body mass index; TG, triglyceride; LDL-C, low-density lipoprotein cholesterol; HDL-C, high-density lipoprotein cholesterol; eGFR, estimated glomerular filtration rate.

Table S3. The association between overall prediabetes, IFG, IGT and elevated HbA1c and increased baPWV in adults with different age and hypertensive status

|  | Normotensive | | | Hypertensive | | |
| --- | --- | --- | --- | --- | --- | --- |
|  | Age <60 years (n=1608) | Age ≥60 years (n=482) | P for interaction | Age <60 years (n=1496) | Age ≥60 years (n=1149) | P for interaction |
| NGR | 1.00 | 1.00 | - | 1.00 | 1.00 | - |
| Prediabetes | 1.90 (1.16-3.11) | 1.04 (0.60-1.78) | 0.073 | 1.11 (0.75-1.65) | 0.83 (0.60-1.16) | 0.084 |
| IFG | 2.04 (1.09-3.80) | 1.15 (0.54-2.44) | 0.131 | 1.60 (1.04-2.47) | 0.97 (0.66-1.43) | 0.065 |
| IGT | 2.07 (1.16-3.70) | 0.44 (0.21-0.95) | <0.001 | 1.06 (0.68-1.64) | 0.66 (0.46-0.93) | 0.020 |
| Elevated HbA1c | 2.14 (1.31-3.47) | 1.16 (0.68-1.99) | 0.076 | 1.05 (0.71-1.55) | 1.13 (0.82-1.56) | 0.659 |

The ORs of prediabetes were calculated compared to normal glucose regulation. The ORs of IFG were calculated compared to normal FPG, and those of IGT were estimated compared to normal 2h-PPG, and those of elevated HbA1c were calculated compared to normal HbA1c. Models were adjusted for age, sex, BMI, TG, LDL-C, HDL-C, uric acid, eGFR, smoking, drinking and anti-hypertensive drug. OR, odds ratio; CI, confidence interval; ROR, ratio of odds ratio; baPWV, branchial-ankle pulse wave velocity; IFG, impaired fasting glucose; IGT, impaired glucose tolerance; HbA1c, glycated hemoglobin; SBP, systolic blood pressure; DBP, diastolic blood pressure; BMI, body mass index; TG, triglyceride; LDL-C, low-density lipoprotein cholesterol; HDL-C, high-density lipoprotein cholesterol; eGFR, estimated glomerular filtration rate.

Table S4. The association between overall prediabetes, IFG, IGT and elevated HbA1c and increased baPWV in adults with different age and obesity status

|  | BMI<24 kg/m2 | | | BMI ≥24 kg/m2 | | |
| --- | --- | --- | --- | --- | --- | --- |
|  | Age <60 years (n=1313) | Age ≥60 years (n=623) | P for interaction | Age <60 years (n=1792) | Age ≥60 years (n=1011) | P for interaction |
| NGR | 1.00 | 1.00 | - | 1.00 | 1.00 | - |
| Prediabetes | 1.05 (0.62-1.78) | 0.82 (0.53-1.26) | 0.146 | 1.55 (1.05-2.30) | 0.97 (0.66-1.44) | 0.054 |
| IFG | 1.30 (0.62-2.74) | 0.83 (0.46-1.47) | 0.120 | 1.79 (1.18-2.70) | 1.10 (0.71-1.71) | 0.090 |
| IGT | 0.68 (0.31-1.50) | 0.48 (0.28-0.83) | 0.162 | 1.58 (1.05-2.37) | 0.71 (0.48-1.06) | 0.002 |
| Elevated HbA1c | 1.26 (0.74-2.15) | 1.09 (0.71-1.66) | 0.384 | 1.47 (1.01-2.13) | 1.13 (0.79-1.63) | 0.197 |

The ORs of prediabetes were calculated compared to normal glucose regulation. The ORs of IFG were calculated compared to normal FPG, and those of IGT were estimated compared to normal 2h-PPG, and those of elevated HbA1c were calculated compared to normal HbA1c. Models were adjusted for age, sex, SBP, DBP, TG, LDL-C, HDL-C, uric acid, eGFR, smoking, drinking and anti-hypertensive drug. OR, odds ratio; CI, confidence interval; ROR, ratio of odds ratio; baPWV, branchial-ankle pulse wave velocity; IFG, impaired fasting glucose; IGT, impaired glucose tolerance; HbA1c, glycated hemoglobin; SBP, systolic blood pressure; DBP, diastolic blood pressure; BMI, body mass index; TG, triglyceride; LDL-C, low-density lipoprotein cholesterol; HDL-C, high-density lipoprotein cholesterol; eGFR, estimated glomerular filtration rate.

Table S5. The association between overall prediabetes, IFG, IGT and elevated HbA1c and increased baPWV in adults with different age and TyG index

|  | TyG index< 4.64 | | | TyG index≥ 4.64 | | |
| --- | --- | --- | --- | --- | --- | --- |
|  | Age <60 years (n=1598) | Age ≥60 years (n=801) | P for interaction | Age <60 years (n=1507) | Age ≥60 years (n=833) | P for interaction |
| NGR | 1.00 | 1.00 | - | 1.00 | 1.00 | - |
| Prediabetes | 1.29 (0.83-1.99) | 1.12 (0.76-1.64) | 0.374 | 1.47 (0.94-2.31) | 0.66 (0.43-1.00) | 0.002 |
| IFG | 2.43 (1.39-4.24) | 1.19 (0.70-2.02) | 0.022 | 1.42 (0.89-2.27) | 0.87 (0.54-1.39) | 0.136 |
| IGT | 0.98 (0.52-1.83) | 0.77 (0.48-1.23) | 0.371 | 1.57 (1.02-2.44) | 0.48 (0.31-0.74) | <0.001 |
| Elevated HbA1c | 1.50 (0.97-2.33) | 1.28 (0.87-1.87) | 0.461 | 1.31 (0.86-2.00) | 1.00 (0.67-1.50) | 0.116 |

The median of TyG index (4.64) was used to divide the subgroups. The ORs of prediabetes were calculated compared to normal glucose regulation. The ORs of IFG were calculated compared to normal FPG, and those of IGT were estimated compared to normal 2h-PPG, and those of elevated HbA1c were calculated compared to normal HbA1c. Models were adjusted for age, sex, SBP, DBP, BMI, LDL-C, HDL-C, uric acid, eGFR, smoking, drinking and anti-hypertensive drug. OR, odds ratio; CI, confidence interval; ROR, ratio of odds ratio; baPWV, branchial-ankle pulse wave velocity; IFG, impaired fasting glucose; IGT, impaired glucose tolerance; HbA1c, glycated hemoglobin; TyG index, triglyceride-glucose index; SBP, systolic blood pressure; DBP, diastolic blood pressure; BMI, body mass index; TG, triglyceride; LDL-C, low-density lipoprotein cholesterol; HDL-C, high-density lipoprotein cholesterol; eGFR, estimated glomerular filtration rate.

Table S6. Adjusted cross-lagged standard regression coefficient of baPWV and FPG

|  | R^2^ of PWV | R^2^ of FBG | baPWV1→baPWV2 | FPG1→FPG2 | FPG1→baPWV2 | baPWV1→FPG2 | P value* |
| --- | --- | --- | --- | --- | --- | --- | --- |
| Age ≥60 | 0.46 | 0.31 | 0.63 (0.61-0.65) | 0.53 (0.51-0.55) | 0.00 (-0.02-0.02) | 0.05 (0.02-0.08) | 0.038 |
| Age <60 | 0.55 | 0.34 | 0.64 (0.63-0.65) | 0.55 (0.54-0.56) | 0.03 (0.02-0.04) | 0.00 (-0.02-0.02) | 0.090 |

The population of sensitivity analysis further excluded those with baseline baPWV higher than the 95th percentile. Model was adjusted for baseline age (quartile), sex, systolic blood pressure (quartile), diastolic blood pressure (quartile), body mass index (quartile), triglyceride (quartile), low-density lipoprotein cholesterol (quartile), high-density lipoprotein cholesterol (quartile), estimated glomerular filtration rate (quartile), uric acid (quartile), current smoking, current drinking, and anti-hypertensive drug. The P value is calculated by t-test of the two cross-lagged standard regression coefficients of baPWV and FPG. BaPWV, branchial-ankle pulse wave velocity; FPG, fasting plasma glucose.

Table S7. Adjusted cross-lagged standard regression coefficient of baPWV and 2h-PPG

|  | R^2^ of PWV | R^2^ of PPG | baPWV1→baPWV2 | PPG1→PPG2 | PPG1→baPWV2 | baPWV1→PPG2 | P value* |
| --- | --- | --- | --- | --- | --- | --- | --- |
| Age ≥60 | 0.46 | 0.28 | 0.63 (0.61-0.65) | 0.46 (0.44-0.48) | -0.04 (-0.06-(-0.02)) | 0.04 (0.01-0.07) | 0.001 |
| Age <60 | 0.55 | 0.27 | 0.65 (0.64-0.66) | 0.46 (0.44-0.48) | -0.01 (-0.02-0.00) | 0.04 (0.02-0.06) | 0.013 |

The population of sensitivity analysis further excluded those with baseline baPWV higher than the 95th percentile. Model was adjusted for baseline age (quartile), sex, systolic blood pressure (quartile), diastolic blood pressure (quartile), body mass index (quartile), triglyceride (quartile), low-density lipoprotein cholesterol (quartile), high-density lipoprotein cholesterol (quartile), estimated glomerular filtration rate (quartile), uric acid (quartile), current smoking, current drinking, and anti-hypertensive drug. The P value is calculated by t-test of the two cross-lagged standard regression coefficients of baPWV and 2h-PPG. BaPWV, branchial-ankle pulse wave velocity; 2h-PPG, 2h-postload plasma glucose.

Table S8. Adjusted cross-lagged standard regression coefficient of baPWV and HbA1c

|  | R^2^ of PWV | R^2^ of HbA1c | baPWV1→baPWV2 | HbA1c1→HbA1c2 | HbA1c1→baPWV2 | baPWV1→HbA1c2 | P value* |
| --- | --- | --- | --- | --- | --- | --- | --- |
| Age ≥60 | 0.46 | 0.39 | 0.63 (0.61-0.65) | 0.59 (0.57-0.61) | 0.03 (0.01-0.05) | 0.04 (0.02-0.06) | 0.364 |
| Age <60 | 0.55 | 0.34 | 0.64 (0.63-0.65) | 0.54 (0.53-0.55) | 0.03 (0.02-0.04) | 0.01 (-0.01-0.03) | 0.187 |

The population of sensitivity analysis further excluded those with baseline baPWV higher than the 95th percentile. Model was adjusted for baseline age (quartile), sex, systolic blood pressure (quartile), diastolic blood pressure (quartile), body mass index (quartile), triglyceride (quartile), low-density lipoprotein cholesterol (quartile), high-density lipoprotein cholesterol (quartile), estimated glomerular filtration rate (quartile), uric acid (quartile), current smoking, current drinking, and anti-hypertensive drug. The P value is calculated by t-test of the two cross-lagged standard regression coefficients of baPWV and HbA1c. BaPWV, branchial-ankle pulse wave velocity; HbA1c, glycated hemoglobin.
